# Preheated Composite as an Alternative for Bonding Feldspathic and Hybrid Ceramics: A Microshear Bond Strength Study

**DOI:** 10.3290/j.jad.b4279775

**Published:** 2023-08-08

**Authors:** Manassés Tercio Vieira Grangeiro, Camila da Silva Rodrigues, Natalia Rivoli Rossi, Karina Barbosa Souza, Renata Marques de Melo, Marco Antonio Bottino

**Affiliations:** a PhD Candidate, Graduate Program in Restorative Dentistry, Institute of Science and Technology, São Paulo State University (UNESP) São José dos Campos, SP, Brazil; Professor, School of Dentistry, Anhanguera University, São José dos Campos, SP, Brazil. Conceptualization, study design, performed experiments, data analysis, wrote the manuscript.; b Post-doctoral Fellow, Institute of Science and Technology, São Paulo State University (UNESP) São José dos Campos, SP, Brazil. Professor, Federal University of Pelotas (UFPel), Pelotas, RS, Brazil. Study design, statistical analysis, wrote the manuscript.; c PhD Student, Graduate Program in Restorative Dentistry, Institute of Science and Technology, São Paulo State University (UNESP) São José dos Campos, SP, Brazil. Performed the experiments, proofread the manuscript.; d MSc Student, Graduate Program in Restorative Dentistry, Institute of Science and Technology, São Paulo State University (UNESP) São José dos Campos, SP, Brazil. Visualization, proofreading.; e Professor, Institute of Science and Technology, São Paulo State University (UNESP) São José dos Campos, SP, Brazil. Supervision, data curation, proofreading.

**Keywords:** bond strength, dental ceramics, adhesion

## Abstract

**Purpose:**

To evaluate the bond strength between alternative or conventional luting agents and indirect restorative materials.

**Materials and Methods:**

Blocks of a polymer-infiltrated ceramic network (PICN, Vita Enamic) and a feldspathic ceramic (FEL, Vita Mark II) were sliced and divided according to the luting agent: resin cement (PICN-RC, FEL-RC), flowable composite (PICN-FC, FEL-FC), or preheated composite (PICN-PH, FEL-PH). The ceramic surfaces were polished, etched with 5% hydrofluoric acid for 60 s, and then a silane layer was applied. Cylinders of the luting agents were built up on the ceramic surfaces. In half the samples, the microshear bond strength (µSBS) was tested after 24 h (baseline). The other half was tested after 5000 thermocycles (5ºC–55ºC) (aging). The failure modes were determined using a stereomicroscope, and the ceramic surfaces were analyzed using a scanning electron microscope. Data were statistically analyzed with two-way ANOVA.

**Results:**

Thermocycling reduced the bond strength values of all experimental groups. Regarding FEL, the preheated composite obtained the highest results. Resin cement showed results similar to the flowable composite at baseline and after aging. The highest results of PICN were obtained from the preheated composite followed by resin cement and flowable composite. Significant differences among the three luting agents were observed before and after aging. The most frequent failures among the experimental groups were adhesive and cohesive in the ceramic.

**Conclusion:**

Bond strength results indicate that the preheated composite can be an alternative for adhesive cementation when applied on the tested feldspathic ceramic or PICN.

Adhesive cementation of feldspathic porcelains and glass-ceramics is paramount to ensure better mechanical behavior and stress distribution along the restoration and substrate.^3,16^ The gold standard protocol for these materials is a combination of mechanical and chemical treatments: first, hydrofluoric acid etching removes the surface glass matrix, leading to silica exposure; then, a silane layer provides chemical adhesion by joining silica from the ceramic and the resin matrix from the cement.^2^

Feldspathic porcelains have been used as an esthetic alternative for veneering metal and polycrystalline ceramic copings. Furthermore, pressing and CAD/CAM methods enable the preparation of feldspathic porcelains with fewer internal defects, which increased their range of applications as a monolith. Since then, review articles have reported high survival rates, eg, 87% for feldspathic porcelain veneers in ~9 years^18^ and 90% for inlays in 5 years.^19^ Aiming for similar clinical applications (onlays, inlays, veneers, single crowns), the polymer-infiltrated ceramic network (PICN) was developed to combine characteristics such as the resilience of resin composites and the wear resistance of ceramics. Hence, both feldspathic and PICN materials require the same cementation protocol (hydrofluoric acid etching and silane).^5,11^

Besides resin cements, flowable resin composites have been studied as an alternative for ceramic luting.^12,15^ However, luting agents with low elastic modulus, for instance, flowable composites, can reduce the strength of dental ceramics,^1^ which could ultimately affect their clinical longevity. Another trend is to preheat resin composites to reduce their viscosity and enable use for cementation of indirect restorations.^13^ Conventional resin composites could perform better than resin cement due to the higher amount of inorganic filling, which would lead to better color stability and mechanical behavior.^13^ However, little information is available on the adhesive behavior of these bonding alternatives, especially on PICN materials.

In this sense, our study aimed to compare the bond strength between alternative luting agents (flowable or preheated conventional resin composites) and indirect restorative materials (felspathic ceramic or PICN), comparing it to a resin cement. In addition, bond strength was evaluated after cementation and after thermocycling aging to observe the long-term adhesive behavior. The tested hypotheses were that (1) alternative and conventional (resin cement) luting agents would lead to similar bond strength to both restorative materials; (2) aging via thermocycling would decrease the bond strength of all experimental groups.

## Materials and Methods

### Study Design

This in-vitro study evaluated two factors – luting agent (resin cement, flowable composite, or preheated regular composite) and aging (baseline or aging) – separately for each restorative material (feldspathic ceramic or PICN). The analyzed outcome was microshear bond strength (µSBS). The commercial brands, manufacturers, and compositions of the materials used in this study are described in Table 1.

**Table 1:** Commercial brands, manufacturers, and compositions of the materials used in this study

Material	Trademark	Manufacturer	Composition
Hybrid ceramic (PICN)	Vita Enamic	Vita Zahnfabrik	86 wt% feldspathic ceramic: SiO_2_ 58–63%, Al_2_O_3_ 20–23%, Na_2_O 9–11%, K_2_O 4–6%; 14 wt%: TEG-DMA, UDMA
Feldspathic ceramic (FEL)	Vita Mark II	Vita Zahnfabrik	20–23% Al_2_O_3_, 6–9% Na_2_O, 6–8% K_2_O, 0.01% TiO_2_, 56–64% SiO_2_, 0.3–0.6% CaO
Hydrofluoric acid	Vita Adiva cera-etch	Vita Zanhfabrik	5% hydrofluoric acid
Silane	Monobond N	Ivoclar Vivadent	Alcoholic solution of silane methacrylate, phosphoric acid methacrylate and sulphide methacrylate
Resin Cement (RC)	Multilink N	Ivoclar Vivadent	Dimethacrylate and HEMA, inorganic particles (barium glass, ethereber trifluoride, and mixed spheroidal oxides)
Flowable composite (FC)	Tetric N Ceram	Ivoclar Vivadent	Bis-GMA, bis-EMA, UDMA, barium glass, ytterbium trifluoride, mixed oxide, silicon dioxide, prepolymers
Preheated composite (PH)	Filtek P60	3M Oral Care	Bis-GMA, UDMA, bis-EMA

Bis-EMA: bisphenol A ethylmethacrylate; bis-GMA: bisphenol A glycidyldimethacrylate; TEG-DMA: triethylene glycol dimethacrylate; UDMA: urethane dimethacrylate.

### Specimens Preparation

**Fig 1:**
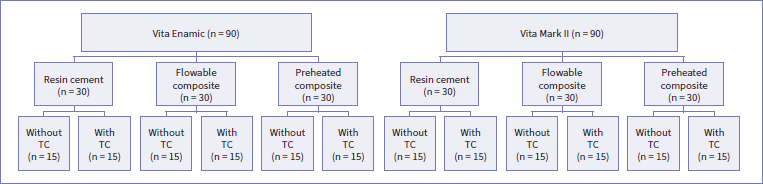
Flowchart showing the experimental groups. TC: thermocycling aging.

Blocks of a polymer-infiltrated ceramic network (PICN, Vita Enamic, Vita Zahnfabrik; Bad Säckingen, Germany) and a feldspathic porcelain (FEL, Vita Mark II, Vita Zahnfabrik) were cut into plates with a diamond saw in a cutting machine (IsoMet 1000, Buehler; Lake Bluff, IL, USA). All the plates were polished with silicon carbide (SiC) papers of #400, #600, and #1200 grit under water cooling in a polishing machine (EcoMet / AutoMet 250, Buehler). The final plates (N = 180 of each material) had dimensions of 10 mm x 8 mm x 2 mm. These were embedded in a chemically activated acrylic resin (JET, Dental Articles Classic; Curitiba, Brazil) as follows: The to-be-treated ceramic was completely cover by a piece of sticky tape. Then, with the non-tape-covered side of the ceramic plate facing up, a 2 cm-high polyvinyl chloride (PVC) cylindrical tube was placed over the taped ceramic, keeping the ceramic plate in the center of the cylinder’s lumen. After that, the tube was filled with acrylic resin. The acrylic resin was cured, and the sticky tape was removed from the resulting PVC cylinder with the ceramic plate in its center. Subsequently, the exposed ceramic surfaces were cleaned with ethanol and subjected to 5-min polishing with #1200 SiC paper to ensure complete removal of the sticky tape. The specimens were randomly divided into 12 groups (n = 15), as shown in Fig 1.

The ceramic surfaces of both materials were etched with 5% hydrofluoric acid for 60 s (Vita adiva cera-etch, Vita Zahnfabrik). Then, the samples were ultrasonically cleaned in distilled water for 5 min. After air drying, a silane layer (Monobond N silane, Ivoclar Vivadent; Schaan, Liechtenstein) was actively applied on the ceramic surfaces with a disposable brush for 10 s and let dry for 60 s.

### Preparation of Luting-Agent Cylinders

After surface treatment, Tygon tubes (Ø = 1.6 mm, height = 2 mm) were placed on each ceramic surface and fixed with wax. For the resin cement groups (PICN-RC and FEL-RC), the luting agent (Multilink N, Ivoclar Vivadent) was inserted into the tubes with a fine tip and photoactivated with an LED curing unit for 40 s (Valo, Ultradent; South Jordan, UT, USA). For the flowable composite groups (PICN-FC and FEL-FC; Tetric N Ceram, Ivoclar Vivadent), the luting agent was inserted into the tubes with the manufacturer’s fine tips and photoactivated using the LED curing unit for 40 s. The resin composite (PICN-PH and FEL-PH) (Filtek P60, 3M Oral Care; St Paul, MN, USA) was preheated in an oven at 68ºC for 10 min, inserted into the tubes with spatula and fine instruments, and photoactivated with the LED curing unit for 40 s. One previously trained operator carried out all the bonding procedures to avoid bias.

### Thermocycling (Simulated Aging)

After the bonding procedures, half of the samples (n = 15) were kept in distilled water at 37°C (Fanem, Orion Estufa de cultura 502; São Paulo, SP, Brazil), for 24 h and then subjected to the microshear bond strength test (baseline testing). The other half (aging testing) were subjected to thermocycling for 5000 cycles in a thermocycling machine (Biopdi, termocycle; São Paulo, SP, Brazil). The temperature of the two water baths were set at 5°C (± 1) and 55°C (± 1) with a dwell time of 30 s. After aging, the specimens were subjected to the μSBS test.

### Microshear Bond Strength (µSBS) Test

The microshear bond strength test was performed in a universal testing machine using a load cell of 50 KgF. The shear load was applied perpendicularly to the cylinder/ceramic interface with an orthodontic wire (Ø = 0.2 mm) at a crosshead speed of 0.5 mm/min until failure. The bond strengths were obtained using the equation R = F/A, where R = bond strength (MPa), F = load to failure (N), and A = interface area (mm^2^). The circular interface area was calculated with the equation A = πr^2^, where π = 3.14 and r is the radius of the resin cement cylinder (0.8 mm). The resulting cross-sectional bonding area was 2 mm^2^.

### Failure Mode Analysis

The tested ceramic samples were inspected using a stereomicroscope (Stereo Discovery V20, Zeiss; Gottingen, Germany) to determine the failure modes: adhesive: failure occurred at the interface between ceramic and RC; cohesive: failure occurred within the RC or the ceramic material; predominantly adhesive: more than 60% of the failure area occurred at the interface between the ceramic and RC.^12^ Representative samples of each observed failure mode were selected and analyzed in a scanning electron microscope (SEM, Vega 3, Tescan; Brno, Czech Republic) at 15 kV and a magnification of 130X.

### Topographic Analysis

PICN and FEL plates were examined using SEM before and after hydrofluoric acid etching to characterize their topographic features (n = 1). The samples were ultrasonically cleaned with ethanol, air dried, sputter-coated with gold, and evaluated using 15 kV and a magnification of 3000X.

### Statistical Analysis

The statistical analysis was performed using Minitab 17 statistical software (Minitab, LLC; State College, PA, USA). The normality and homoscedasticity of the µSBS data were checked by Shapiro-Wilk and Levene’s tests, respectively. Then, a two-way ANOVA was carried out (factors luting agent and aging) separately for FEL or PICN results. Multiple comparison analyses were performed using Tukey’s test. The significance level was set at 5%.

## Results

### Microshear Bond Strength

The µSBS means and standard deviations are given in Table 2. The factors luting agent and aging significantly affected the bond strength of both feldspathic and PICN materials (p < 0.001 for all factors). The interactions between luting agent and aging were not significant (PICN: p = 0.176; FEL: p = 0.261), meaning that the luting agent effect does not depend on aging.

**Table 2:** Means (standard deviations) of microshear bond strength (MPa) obtained from each experimental group

Ceramic material	Luting agent	Baseline	Aging
PICN	RC	27.5 (3.5)^Ab^	22.2 (3.2)^Bb^
	FC	22.9 (4.1)^Ac^	16.6 (3.3)^Bc^
	PH	33.4 (4.1)^Aa^	25.6 (3.4)^Aa^
FEL	RC	23.1 (5.2)^Ab^	17.3 (4.3)^Bb^
	FC	21.4 (3.3)^Ab^	15.8 (5.2)^Bb^
	PH	25.2 (3.9)^Aa^	21.8 (4.7)^Ba^

Different superscript uppercase letters within a row indicate statistically significant differences between baseline and aging for each group. Different superscript lowercase letters within a column indicate significant differences between the experimental groups at baseline or after aging separately for each restorative material (two-way ANOVA, Tukey’s test, p < 0.05).

Thermocycling reduced the bond strengths of all experimental groups. Regarding the feldspathic ceramic, the preheated composite reached the highest bond strengths; the µSBS of the RC and FC were both lower than that of the preheated composite but similar to each other. These results were observed at baseline and after aging. For PICN, significant differences among the three luting agents were observed before and after aging. The preheated composite produced the highest bond strengths, followed by resin cement and flowable composite.

### Failure Modes

**Fig 2:**
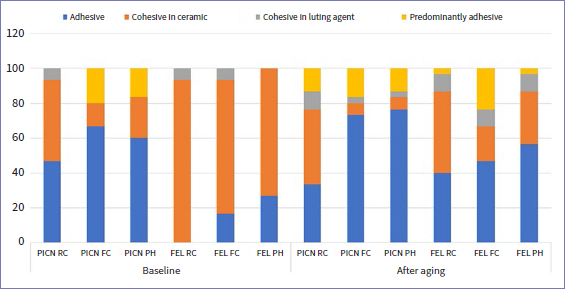
Failure mode distribution. FEL: feldspathic porcelain, PICN: polymer-infiltrated ceramic network, RC: resin cement, FC: flowable composite, PH: preheated composite.

**Fig 3:**
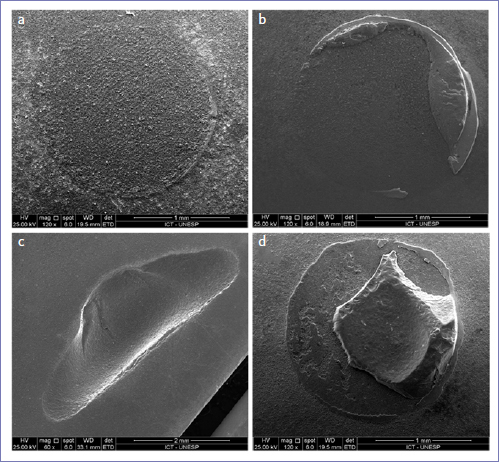
SEM images of representative samples showing the observed failure modes: adhesive (a); predominantly adhesive (b); cohesive in ceramic (c); and cohesive in resin cement (d). Original magnification: 60 to 120X.

Figure 2 depicts the failure mode distribution among the experimental groups. The most frequent failures were adhesive and cohesive in ceramic. Among the FEL groups, a higher number of cohesive failures was observed at baseline, whereas adhesive failures were more frequently observed after aging. A similar pattern was observed before and after aging among PICN groups. Representative images of adhesive, predominantly adhesive, cohesive in ceramic, and cohesive in resin cement are illustrated in Fig 3.

### Topographic Analysis

**Fig 4:**
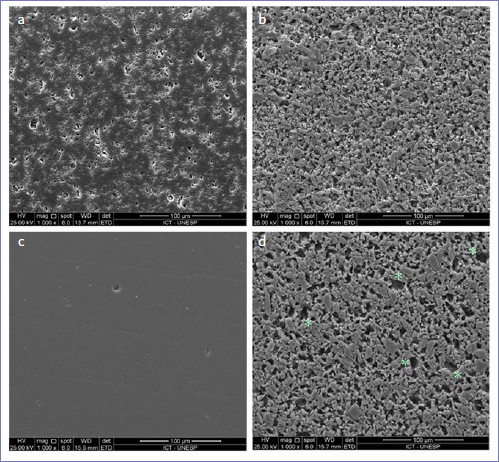
SEM surface images of the tested materials: PICN before (a) and after (b) hydrofluoric acid etching; FEL before (c) and after (d) etching. Larger defects* caused by the acid etching are visible on the feldspathic ceramic surface (d).

Figure 4 shows the surface images taken from PICN and FEL before and after hydrofluoric acid etching. These images demonstrate the surface modification caused by hydrofluoric acid. SEM inspection revealed greater size defects on the feldspathic porcelain when compared to PICN after etching (Figs 4b and 4d).

## Discussion

This study compared the bond strength between alternative luting agents and a feldspathic ceramic or a polymer-infiltrated ceramic network material. The preheated composite yielded the highest bond strengths on PICN and FEL, even after aging. In addition, resin cement and flowable composite yielded similar results on FEL. On PICN, the flowable composite yielded the lowest bond strengths, especially after aging. These findings led to rejection of the first tested hypothesis, since the different luting agents mediated different bond strengths.

Our results pointed out that the luting agent effect depends on the ceramic material. Despite being a PICN material, the surface polymeric matrix is removed by hydrofluoric acid etching, which results in a topography similar that of an acid-etched feldspathic ceramic (Fig 4). Nonetheless, the SEM analysis revealed a higher number of larger defects on the feldspathic ceramic surface than on PICN. It is well known that the surface modification caused by hydrofluoric acid is vital for long-term adhesion between glass-ceramics and resin cements.^4,17^ However, the topographic differences observed between PICN and FEL could have affected the luting agents’ interlocking, which ultimately led to different bond strengths.

The flowable composite and resin cement have less inorganic filler particles in their composition, which is one reason these materials are less viscous.^12,14^ Fluid luting agents are expected to penetrate better into the surface irregularities of acid-etched ceramic materials. Nevertheless, less viscous composites tend to shrink more after polymerization due to the low amount of filler particles.^9^ The polymerization shrinkage of the flowable composite, together with the easier solubility of these materials,^10^ resulted in the lowest bond strengths observed in this study. In contrast, resin composite tends to shrink less due to the higher amount of filler particles, as demonstrated in previous studies.^8^ Once the resin composite is heated and the viscosity decreases, it spreads into the ceramic surface irregularities.^6^ After the composite polymerizes, better interlocking is expected due to the lower shrinkage. Hence, when the preheated composite was applied over a surface with smaller defects (PICN), higher bond strengths were observed (~33 MPa).

The bond strength of all experimental groups decreased after thermocycling, so that the second tested hypothesis was accepted. Composites experience hydrolytic degradation due to water sorption,^7^ which explains the bond strength drop observed in all experimental groups. In addition, composites with less filler particles tend to degrade faster in water.^7^ This was demonstrated by the low bond strengths yielded by the flowable composite. The greater size defects on FEL could have contributed to the bond strength decrease in RC and FC groups, since they create areas with thicker layers of these low particle-filled materials which tend to degrade more easily. However, it should be noted that these results were obtained from a variable-controlled in-vitro study. The difference between adhesive cementation with traditional resin cement or preheated composite must be explored in a more clinically relevant approach.

The microshear bond strength test was chosen due to its easier sample preparation and testing compared to (micro)tensile set-ups. We built up cylinders with 2 mm^2^ area, which reduces the probability of introducing defects at the interface. Moreover, the failure modes observed in our study were mostly adhesive or cohesive in ceramic, which are in agreement with the failure modes reported in previous bond strength studies on glass-ceramics or PICN.^4,17^ The use of bond strength tests to predict clinical behavior is questionable, because it is challenging to establish a correlation between laboratory data and clinical performance. Even so, these types of tests are important for observing differences between bonding strategies and selecting those that merit further evaluation in clinical studies.

The preheated composite achieved the highest bond strengths when used on PICN or feldspathic porcelain even after thermocycling. These results suggest that preheated composite may be a suitable alternative for bonding PICN and feldspathic ceramics. Nevertheless, more studies are necessary to investigate other aspects of using preheated composites as luting agents, such as marginal adaptation, optical properties, and mechanical behavior. In addition, similar results were obtained from resin cement and flowable composite on the feldspathic ceramic. All of the above-mentioned aspects must be considered before choosing flowable composite as a luting agent.

## Conclusion

The present bond strength results indicate that preheated composite can be an alternative for adhesive cementation when applied on the tested feldspathic ceramic or PICN. However, the use of a flowable composite for cementation of PICN is not encouraged, due to the low bond strengths observed in this study.

## Clinical relevance:

Regarding bond strength, preheated conventional composite can be an alternative for bonding feldspathic and hybrid ceramics. However, compared to resin cement, flowable composite can lead to lower adhesion.
